# Social rejection sensitivity and its role in adolescent emotional disorder symptomatology

**DOI:** 10.1186/s13034-022-00555-x

**Published:** 2023-01-16

**Authors:** Savannah Minihan, Cassandra Kwok, Susanne Schweizer

**Affiliations:** 1 Developmental Affective Science Lab, School of Psychology, University of New South Wales, Kensington, Australia; 2 Developmental Cognitive Neuroscience Group, Department of Psychology, University of Cambridge, Cambridge, UK

**Keywords:** Social rejection sensitivity, Interpretation bias, Parental rejection, Emotional disorders, Adolescent mental health

## Abstract

**Background:**

Most emotional disorders first emerge during adolescence, a time characterized by heightened sensitivity to social information, especially social rejection. Social rejection sensitivity (SRS), then, may be a promising intervention target.

**Methods:**

To explore this, 357 participants (*M* (*SD*) age = 19.40 (4.18), 63% female) completed self-report measures of SRS, its proposed antecedent, perceived parenting style, its proposed behavioral correlate, negative interpretation bias, and its proposed  clinical correlate, emotional disorder symptoms. Participants additionally completed a single session of a social interpretation bias modification task, the ambiguous social scenarios task (ASST).

**Results:**

SRS was associated with perceived parental rejection, while controlling for other types of maladaptive parenting. SRS partially accounted for variance in the relationship between perceived parental rejection and emotional disorder symptomatology, as well as the relationship between negative interpretation bias and emotional disorder symptoms. Learning rates (i.e., change in reaction time across the task) on the ASST differed as a function of age and SRS, such that younger participants with higher SRS showed the slowest rate of learning. Moreover, individual differences in SRS accounted for the magnitude of change in negative interpretation bias before and after the ASST. Individuals with greater SRS showed *less* change in interpretation bias.

**Conclusions:**

SRS appears strongly associated with emotional disorder symptoms in adolescents. Importantly, SRS was associated with the malleability of negative interpretation bias, which may help account for the mixed findings on the effectiveness of interpretation-bias-modification-paradigms in adolescents.

**Supplementary Information:**

The online version contains supplementary material available at 10.1186/s13034-022-00555-x.

75% of all mental health disorders [1] emerge before the end of adolescence (10–24 years [2]). Rapee and colleagues [3] have argued that it is in particular emotional disorders (incl., depression and generalized anxiety) that first emerge in adolescence. These disorders are associated with increased negative affect, impaired social functioning and for many they will recur across the lifespan [1]. Optimizing prevention and early intervention for emotional disorders is therefore essential. To-do-so we must first identify malleable risk factors that contribute to their onset [4]. A hypothesized risk factor for emotional disorders is social rejection sensitivity (SRS), a trait characterized by the tendency to anxiously expect, readily perceive, and overreact to social rejection [5]. Given the greater (compared to both adults and children) social sensitivity that characterizes adolescence, the impact of SRS on mental health symptoms and associated outcomes may be heightened amongst adolescents. The current study therefore explored the role of SRS in emotional disorder symptoms, from adolescence to early adulthood.

## Social rejection sensitivity and emotional disorder symptomatology

SRS shows moderate associations with emotional disorder symptoms cross-sectionally and prospectively, in both adolescents and adults [6]. Theoretical models of the role of SRS in emotional disorders have proposed that this trait develops as a consequence of early experiences of rejection [7], whereby the human need for belongingness and acceptance is too often met with rejection. This discrepancy results in a bias toward the anticipation of rejection by others. In situations where being rejected is a possibility, such as ambiguous social situations, these expectations are activated, leading individuals to readily perceive innocuous or ambiguous cues as evidence of rejection [8]. With repeated experiences of (perceived or actual) rejection, SRS can increase over time, resulting in hypersensitivity to cues of rejection by others, behaviorally expressed as a negative interpretation bias in ambiguous social situations [9]. Supporting theoretical models, perceived parental rejection has been associated both cross-sectionally [10–13] and longitudinally [14] with SRS in adolescents. Moreover, there is preliminary, cross-sectional evidence that SRS partially accounts for variance in the association between negative parenting practices (incl., rejection, coercion, and psychological control) and emotional disorder symptomatology in adolescents [13]. High SRS may then be a mechanism through which adverse early parenting impacts later mental health in adolescents.

A proposed cognitive expression of SRS [9, 18] is negative interpretation bias, the tendency to interpret ambiguous situations negatively. Negative interpretation bias is a well-established common feature of emotional disorders in both adolescents and adults [15–17] . Encouragingly, however, converging evidence suggests that negative interpretation bias can be modified via targeted training [19]. Cognitive Bias Modification for Interpretation (CBM-I) requires individuals to repeatedly resolve ambiguous situations in a positive or benign manner. The rationale is that repeated exposure to positive resolutions of ambiguous situations will override prepotent negative interpretation tendencies. Evidence in the adult literature suggests that CBM-I yields small but significant improvements in emotional disorder symptomatology [19, 20]. However, some studies have failed to show an effect of CBM-I in adolescents [21, 22]. Understanding the source of these mixed findings is an important step in optimizing the outcomes of CBM-I. One source of individual differences that may partially account for the mixed findings is SRS. High SRS may be a marker of more ingrained social interpretation bias, thereby limiting the potential to learn novel response tendencies on CBM-I type interventions. Alternatively, individuals with high levels of SRS may be more susceptible to beneficial effects of CBM-I, especially if the CBM-I training focuses on socially ambiguous situations. Understanding the potential of CBM-I to mitigate the detrimental effects of SRS is critical, as there are currently limited, if any, interventions targeting SRS directly.

## The present study

The present study had two overarching aims. First, we aimed to replicate and extend the proposed associations between emotional disorder symptoms and SRS. Our second aim was to investigate whether SRS accounts for variation in the malleability of individuals’ negative interpretation bias. Given the increased social sensitivity that characterizes adolescence, we were additionally interested in investigating age-related variability in the observed relationships. In order to do so, individuals aged from adolescence to early adulthood were included (11–30 years).

To address our first aim, participants completed self-report measures of SRS, negative interpretation bias, emotional disorder symptomatology, and perceived parenting. Together this allowed us to test the prediction that *SRS would be positively correlated with negative interpretation bias and emotional disorder symptomatology* (H1a), and that levels of all three (i.e., SRS, negative interpretation bias, and emotional disorder symptomatology) would *be associated with perceived parenting style*, such that greater perceived parental rejection, behavioural control, and psychological control would be associated with heightened SRS, negative interpretation bias and emotional disorder symptomatology (H1b). Exploring this association further, we predicted that *SRS and negative interpretation bias would partially account for variance in the association between parenting styles and emotional disorder symptomatology* (H2). Moreover, we predicted that *SRS would partially account for variance in the relationship between negative interpretation bias and emotional disorder symptomatology* (H3). Potential age-related variance in the observed relationships was investigated by including age as a covariate.

To address our second aim, we developed a novel CBM-I-like, ambiguous social scenarios task (ASST), on which participants had to learn to resolve ambiguous social scenarios correctly. We predicted that: *Increased SRS, negative interpretation bias* (H4) *and emotional disorder symptoms* (H5) *would be associated with slower learning on the ASST* (i.e., less change in reaction time and accuracy across the task), and that the *magnitude of change in interpretation bias following the task would increase as a function of SRS* (H6). Finally, age was included to investigate whether the impact of SRS on learning rates and the malleability of negative interpretation bias differed as a function of age.

The study method and hypotheses were pre-registered prior to the recruitment of participants (https://osf.io/nwvs9—see methods for a deviation from protocol).

## Methods

### Participants

Participants were 463^1^ individuals, who were recruited via the University of New South Wales research participation system, social media advertising, emails to high schools, and the MQ participate page. To be included in the study, participants had to be fluent in English, be aged 11 to 30 years, live in Australia, the United Kingdom, or the United States, and have no history of traumatic brain injury or neurodevelopmental/neurological disorder. A total of 106 participants had to be excluded (for reasons for exclusion see Additional file 1: 1 ).

The final sample (*N* = 357; 11–30 years, *M* (*SD*) = 19.40 years (4.18 years)) was demographically diverse (Table 1). Just over half identified as female and less than half as White and of high SES. Representative of prevalence rates in the general population (26), 24.37% reported a history of mental health problems.

**Table 1:** Summary of participant characteristics

Participant characteristics	*n* (%)
Age	
11–15 years	51 (14.28%)
16–24 years	231 (64.71%)
25–30 years	55 (15.41%)
Missing	20 (5.60%)
Gender	
Female	226 (63.31%)
Male	122 (34.25%)
Other	6 (1.66%)
Prefer not to say	3 (0.83%)
Ethnicity	
White	174 (48.74%)
Asian	109 (30.53%)
Mixed	27 (7.56%)
African	11 (3.08%)
Hispanic	10 (2.80%)
Aboriginal or Torres Strait Islander	9 (2.52%)
Other	11 (3.08%)
Prefer not to say	6 (1.68%)
Country	
Australia	200 (56.02%)
United States of America	142 (39.78%)
United Kingdom	14 (3.92%)
Missing	1 (0.28%)
SES	
High	178 (49.86%)
Middle – High	33 (9.24%)
Middle	138 (38.66%)
Low—Middle	2 (0.56%)
Low	6 (1.68%)
History of mental health diagnosis	87 (24.37%)

SES = Socioeconomic status; high = university, middle = high school or professional/vocational training, low = primary school. For participants over the age of 18, SES was operationalized as participants highest educational attainment. For participants under the age of 18, SES was operationalized as the average of their parent’s highest educational attainment. Parental education has been shown to be a robust indicator of SES [62]

### Measures

#### Social rejection sensitivity

The Online and Offline Social Sensitivity Scale (O^2^S^3^ [25]) was used to measure SRS. The 18-item scale assesses SRS in both off- and on-line contexts, given that many of today’s social interactions, especially amongst adolescents, occur online. Participants were required to indicate the extent to which such items as “I worry about the effect I have on other people” and “I delete my social media posts if I don’t get the responses I wanted” describe themselves on a 4-point Likert scale ranging from 0 (*Strongly Disagree*) to 3 (*Strongly Agree*). A total score was computed by summing all items, such that higher scores indicate greater SRS. The scale has shown good internal consistency (*ωT* = 0.90 to 0.93) as well as strong associations with symptoms of emotional disorders (*r* = 0.58 [25]). The O^2^S^3^ demonstrated good internal consistency in the current study (*ωT* = 0.93).^2^

#### Interpretation bias

The scrambled sentences task [27] was administered to assess change in negative interpretation bias from pre- to post-ASST. The current version was developed to assess interpretation bias in adolescents [23]. The task comprised 40 statements reflecting general and social-anxiety related concerns. The scrambled sentences consisted of six words, which could be unscrambled using five of the six words to form a positively or negatively connotated statement.^3^ For example, the scrambled sentence “people dislike new enjoy meeting I” could be unscrambled to “I enjoy meeting new people” (positive) or “I dislike meeting new people” (negative). In the current study, we modified one of the sentences to make it age appropriate for our sample. Specifically, the statement, “relaxed with tense I’m *children older*”, was changed to, “relaxed with tense I’m *people other*”. In addition, one neutral sentence was added to each administration of the task (specifically, “lunch time it dinner is for” and “I read like books to magazines”) to provide a baseline response time for this task. Including this as a covariate in analyses did not change the pattern of results.

Participants were shown each sentence on a trial-by-trial basis and asked to click on five of the six words presented to unscramble the sentence. Participants were given 30 s to complete each sentence, with a timer shown on the screen. The task was completed under a cognitive load in order to disrupt volitional efforts to suppress, modify, or edit responses. Cognitive load was introduced by presenting participants with a four-digit number at the start of the task and asking them to keep it in mind to be recalled at the end of the task. Half of the statements were presented immediately prior to completion of the ASST, the remaining half were presented immediately after completion of the task. Presentation order (pre vs. post) of the statements was counterbalanced across participants. Interpretation bias was operationalized as the proportion of sentences completed grammatically correctly with a negative valence, such that higher scores indicated a greater negative interpretation bias [23].

As per our pre-registration, we also administered the Adolescent Interpretation and Belief Questionnaire [28], which is a self-report measure of interpretation bias. However, the questionnaire demonstrated variable internal consistency across subscales in the current study (*ωT* = 0.42 to 0.80). Given such variability, as well as poor internal consistency observed on this measure in previous studies [29], this questionnaire was excluded from analyses. Consequently, negative interpretation bias as measured by the pre-ASST scrambled sentences task was included as the outcome (H1), mediator (H2), and predictor variable (H3 and H4) in H1 to H4.

#### Emotional disorder symptomatology

Emotional disorder symptoms were assessed with the Depression Anxiety and Stress Scale-21 (DASS-21 [30]) and the Strengths and Difficulties Questionnaire (SDQ [31]). On the DASS-21, participants rated the extent to which such items as “I couldn’t seem to experience any positive feeling at all” applied to themselves over the previous week, on a 4-point Likert scale ranging from 0 (*Did not apply to me at all*) to 3 (*Applied to me very much, or most of the time).* A total score was computed by summing all items and multiplying by two (ensuring that scores are on the same range as the DASS-42 as per the scoring protocol for the DASS-21). On the SDQ, participants rated the extent to which such items as “I am often unhappy, depressed, or tearful” were true of themselves in relation to the previous six months, on a scale ranging from 0 (*Not true)* to 2 (*Certainly true).* An internalizing score was computed by summing items on the emotional and peer problems subscales. Total scores on the DASS-21 and internalizing scores on the SDQ were z-transformed and summed, to create a composite emotional disorder symptomatology score. Both questionnaires have good psychometric properties [30–35]. Acceptable internal consistency was observed on both questionnaires in the current study (*ωT* ranged from 0.80 to 0.97).

#### Perceived parenting style

The 24-item PASCQ [36] was used to measure participant’s perceived parenting style. The PASCQ includes six subscales assessing warmth, rejection, structure, chaos, autonomy support, and coercion. Participants rated such items as “Sometimes I wonder if my parents like me” on a 4-point Likert scale, ranging from 1 (*Not at all true)* to 4 (*Very true).* The PASCQ has demonstrated adequate psychometric properties [12, 36, 37]. Indices of three parenting dimensions: rejection*,* psychological control and behavioural control, were computed with the averaged scores on the warmth (reverse-scored) and rejection subscales; autonomy (reverse-scored) and coercion subscales; and structure (reverse-scored) and chaos subscales, respectively. In the current study, the total scale and three derived indices demonstrated good internal consistency (*ωT* ranged from 0.91 to 0.96).

#### Ambiguous social scenarios task

Participants’ responses to ambiguous social scenarios were evaluated using a novel ASST. CBM-I tasks typically require participants to read or listen to a series of stand-alone ambiguous scenarios and solve simple word fragments to disambiguate the scenarios. The ASST was designed to create a more immersive paradigm that would be particularly relatable for adolescents. Specifically, we paired text-based scenarios with related images and linked the scenarios together in a series of seven adolescent-relevant themes (sports match, individual art performance, individual presentation (school), group work, change in appearance, one-on-one messaging, sleepover). Each scenario depicted an ambiguous social interaction in which the scenario’s protagonist could potentially be rejected. Participants were then presented with two possible resolutions for each scenario, one positive and one negative, and asked to indicate which resolution they thought most likely to occur. An example scenario is “You are working on a group project and you have an idea for your presentation that is different to what the others in your group want to do. You tell them your idea”. Participants selected one of two possible resolutions: “Your group ignores your suggestion and keeps working on the project” (negative) or “Your group asks you to explain your suggestion in more detail” (positive). Participants had to learn to resolve these scenarios positively, thereby reducing the prepotent negative interpretation bias evoked by the scenarios in individuals high on SRS. Each theme comprised six sequential scenarios, four of which were socially ambiguous and two of which were neutral. Neutral scenarios were included to motivate task engagement. That is, to ensure that participants read the scenarios before selecting a resolution, as unlike with the socially ambiguous scenarios there was no obvious “correct” response for the neutral scenarios. Presentation order of themes was randomized across participants.

Learning on the task was promoted by introducing ‘social points’. Participants were told that the aim of the task was to collect as many social points as possible, which they were awarded each time they selected the positive resolution. In the neutral trials, resolutions were both neutral and one was randomly selected to be “correct”. Response to the ASST was computed as average response time (for positive resolutions) and response accuracy (proportion of positive resolutions selected) for each theme. Learning rates on the ASST were operationalized as change in reaction time and accuracy across the task, such that a greater decrease in reaction time and greater increase in accuracy across the task indicated greater learning (i.e., learning more quickly to resolve the ambiguous social scenarios correctly (i.e., positively)).

### Procedure

Participants first provided informed consent. For participants under the age of 18 parental consent was also obtained. Next, participants completed all self-report measures and the affective backward digit span task.^4^ Participants then completed the scrambled sentences task, immediately before and after the ASST. Participants were compensated with course credit or AUD $20. Testing sessions lasted approximately one hour and were completed online on the Gorilla testing platform (www.gorilla.sc). The study was approved by the University of New South Wales Human Research Ethics Committee [HC200214].

### Data analysis

General linear models (incl. correlations, linear regression models, and mediation models) were used to investigate the relationship between SRS, negative interpretation bias, emotional disorder symptoms, and perceived parenting style (H1 to H3). Specifically, H1 was investigated with a general linear model including perceived parental rejection, psychological control, and behavioural control as predictors, and separate models were specified for each outcome variable (i.e., SRS, negative interpretation bias, and emotional disorder symptoms). In the first mediation model (H2), perceived parental rejection was included as the predictor variable, SRS and negative interpretation bias as multiple mediators (allowed to covary), and emotional disorder symptoms as the outcome variable. In the second mediation model (H3), negative interpretation bias was included as the predictor, SRS as the mediator, and emotional disorder symptoms as the outcome variable. These mediation analyses do not, however, imply causal or temporal mediation as they were conducted in cross-sectional data.

Before analysing the influence of SRS, negative interpretation bias and emotional disorder symptomatology on learning rates on the ASST (i.e., change in reaction time and accuracy across the task), data on the task were cleaned (see Additional file 1: 2). Due to poor skew and kurtosis on both reaction time and accuracy data after cleaning, the reaction time data were log transformed and the accuracy data were transformed into a binary distribution (1 = 100% correct; 0 = < 100% correct). The influence of SRS, negative interpretation bias (H4), and emotional disorder symptomatology (H5) on learning rates on the ASST was investigated with linear mixed effects models and generalized estimation equation models (for reaction time and accuracy data, respectively). Time (i.e., theme 1 to 7 on the ASST, with the first theme coded as 0), SRS/negative interpretation bias (H4), and emotional disorder symptoms (H5), and their interactions were included as predictor variables, with a random effect for participant ID. Mixed linear effects analyses were also used to investigate whether change in negative interpretation bias measured before and after the ASST would increase as a function of SRS (H6). Time (i.e., from pre- to post-ASST, with pre-ASST coded as 0), SRS, and their interactions were included as predictor variables, with a random effect for participant ID. In order to investigate age-related variance in the effect of SRS on learning rates and the malleability of negative interpretation bias, age was included as an interaction term in these analyses. All continuous variables included in interaction terms were mean-centered.

Our primary emotional disorder measure was a composite score of the z-transformed DASS-21 total score and the z-transformed SDQ internalizing score. However, all relevant analyses (H1 to H3 and H5) were repeated with the DASS-21 depression and anxiety subscales separately, to explore specificity of effects to depression or anxiety. A Bonferroni-corrected significance level of *p* < 0.017 (0.05/3) was applied to these exploratory analyses. The results for these exploratory analyses showed the same pattern observed in our primary analyses (see Additional file 1).

All analyses were conducted in R version 4.1.2 [39]. Correlations were analyzed using the corrplot package [40] and psych package [41]; general linear models were analyzed using the stats package [39]; mediation models were analyzed using the Lavaan package [42]; mixed linear effects models were analyzed using the Afex package [43]; and generalized estimation equation model were analyzed using the geepack package [44]. Figures were made using the interactions package [45] and the ggplot2 package [46].

## Results

### The relationship between social rejection sensitivity, negative interpretation bias, perceived parenting styles, and emotional disorder symptoms

Supporting the first hypothesis, SRS, negative interpretation bias, perceived parenting styles and emotional disorder symptoms all showed moderate to large positive correlations (Table 2). Age was not significantly correlated with any variable of interest.

**Table 2:** Descriptive statistics and correlations between all variables of interest

Variable	*M (SD)*	Range	1	2	3	4	5	6	7	8
SRS	28.92 (10.99)	3:52	1.00							
Interpretation bias	0.46 (0.25)	0:1	0.51***	1.00						
Emotional disorder symptoms	− 0.01 (1.86)	− 3.57:5.28	0.65***	0.64***	1.00					
Parental rejection	1.93 (0.73)	1:4	0.44***	0.38***	0.53***	1.00				
Parental behavioural control	2.22 (0.71)	1:4	0.34***	0.35***	0.48***	0.78***	1.00			
Parental psychological control	2.12 (0.72)	1:4	0.40***	0.38***	0.48***	0.83***	0.78***	1.00		
Affective control	0.04 (0.43)	− 0.73:2.50	− 0.13	− 0.08	− 0.11	− 0.02	− 0.01	− 0.13	1.00	
Age	19.40 (4.18)	11:30	− 0.01	0.01	− 0.03	− 0.08	− 0.09	− 0.06	0.10	1.00

SRS = social rejection sensitivity measured by the total score on the O^2^S^3^ [25]; interpretation bias = negative interpretation bias measured as proportion of grammatically correct sentences with negative valence on the pre-ASST scrambled sentences task [23]; emotional disorder symptoms = emotional disorder symptoms measured as composite score of standardized DASS-21 total score [30] and standardized SDQ internalizing score [31]; parental rejection = perceived parental rejection measured as eponymous subscale score of the PASCQ [36]; parental behavioural control = perceived parental behavioural control measured as eponymous subscale score of the PASCQ [36]; parental psychological control = perceived parental psychological control measured as eponymous subscale score of the PASCQ [36]; affective control = affective control measured as proportional difference score on the affective digit span task [38]. * *p* < 0.05, **p < 0.01, ***p < 0.001. Pearson’s correlations are reported.

Perceived parental rejection was found to be associated with heightened SRS (*b* = 5.90, *SE* = 1.59, *t* = 3.70, *p* < 0.001), negative interpretation bias (*b* = 0.08, *SE* = 0.04, *t* = 2.21, *p* = 0.028), and emotional disorder symptomatology (*b* = 0.99, *SE* = 0.27, *t* = 3.71, *p* < 0.001), while controlling for perceived parental psychological control and behavioural control (H1; Additional file 1: Table S1). Conversely, perceived parental psychological and behavioural control were not associated with SRS, negative interpretation bias, or emotional disorder symptoms, while controlling for perceived parental rejection (*p*’s > 0.117). Given these findings, parental rejection was included as the sole index of perceived parenting style in subsequent analyses.

**Fig. 1:**
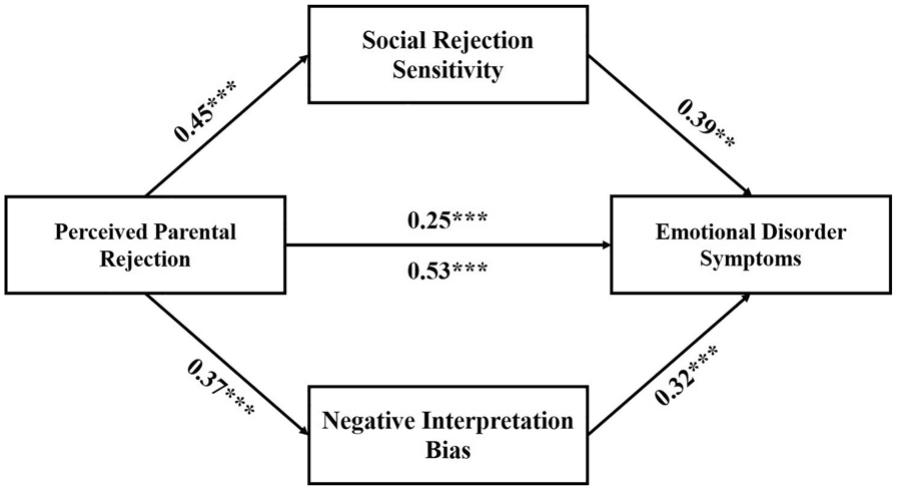
Significant indirect effect of social rejection sensitivity and negative interpretation bias on the relationship between perceived parental rejection and emotional disorder symptoms. Social rejection sensitivity = social rejection sensitivity measured by the total score on the O^2^S^3^ [25]; negative interpretation bias = negative interpretation bias measured as proportion of grammatically correct sentences with negative valence on the pre-ASST scrambled sentences task [23]; emotional disorder symptomatology = emotional disorder symptoms measured as composite score of standardized DASS-21 total score [30] and standardized SDQ internalizing score [31]; perceived parental rejection = perceived parental rejection measured as eponymous subscale score of the PASCQ [36]. The paths include standardized *β* estimates of the associations. The two mediators were allowed to covary. The figure includes the effect of perceived parental rejection on emotional disorder symptoms with (above the arrow) and without (below the arrow) controlling for social rejection sensitivity and negative interpretation bias. Age was included as a covariate in the model but is not depicted for simplicity. *p < 0.05, **p < 0.01, ***p < 0.001.

In line with H2, SRS and negative interpretation bias were found to partially account for variance in the relationship between perceived parental rejection and emotional disorder symptoms, while controlling for age; standardized indirect effect SRS: *β* = 0.18, *SE* = 0.04, *z* = 5.49, *p* < 0.001; standardized indirect effect negative interpretation bias: *β* = 0.12, *SE* = 0.04, *z* = 4.37, *p* < 0.001; Akaike Information Criterion (AIC) = 2131.49 (Fig. 1). That is, heightened perceived parental rejection was associated with heightened social rejection sensitivity and negative interpretation bias, which in turn, were associated with heightened emotional disorder symptoms.

**Fig. 2:**
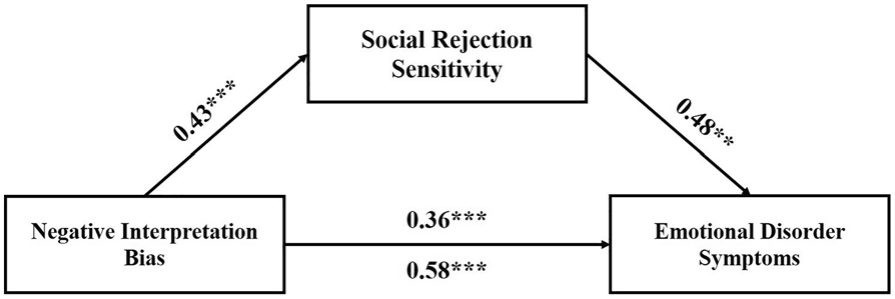
Significant indirect effect of social rejection sensitivity on the relationship between negative interpretation bias and emotional disorder symptoms. Social rejection sensitivity = social rejection sensitivity measured by the total score on the O^2^S^3^ [25]; Negative interpretation bias = negative interpretation bias measured as proportion of grammatically correct sentences with negative valence on the pre-ASST scrambled sentences task [23]; emotional disorder symptomatology = emotional disorder symptoms measured as composite score of standardized DASS-21 total score [30] and standardized SDQ internalizing score [31]. The paths include standardized *β* estimates of the associations. The figure includes the effect of negative interpretation bias on emotional disorder symptoms with (above the arrow) and without (below the arrow) controlling for social rejection sensitivity. Age was included as a covariate in the model but is not depicted for simplicity. *p < 0.05, **p < 0.01, ***p < 0.001.

Again, confirming our predictions (H3), SRS was found to partially account for the relationship between negative interpretation bias and emotional disorder symptoms; standardized indirect effect: *β* = 0.21, SE = 0.13, z = 6.35, *p* < 0.001; AIC = 1378.61 (Fig. 2).

### The impact of social rejection sensitivity, negative interpretation bias, and emotional disorder symptomatology on learning rates on the ambiguous social scenarios task

**Fig. 3:**
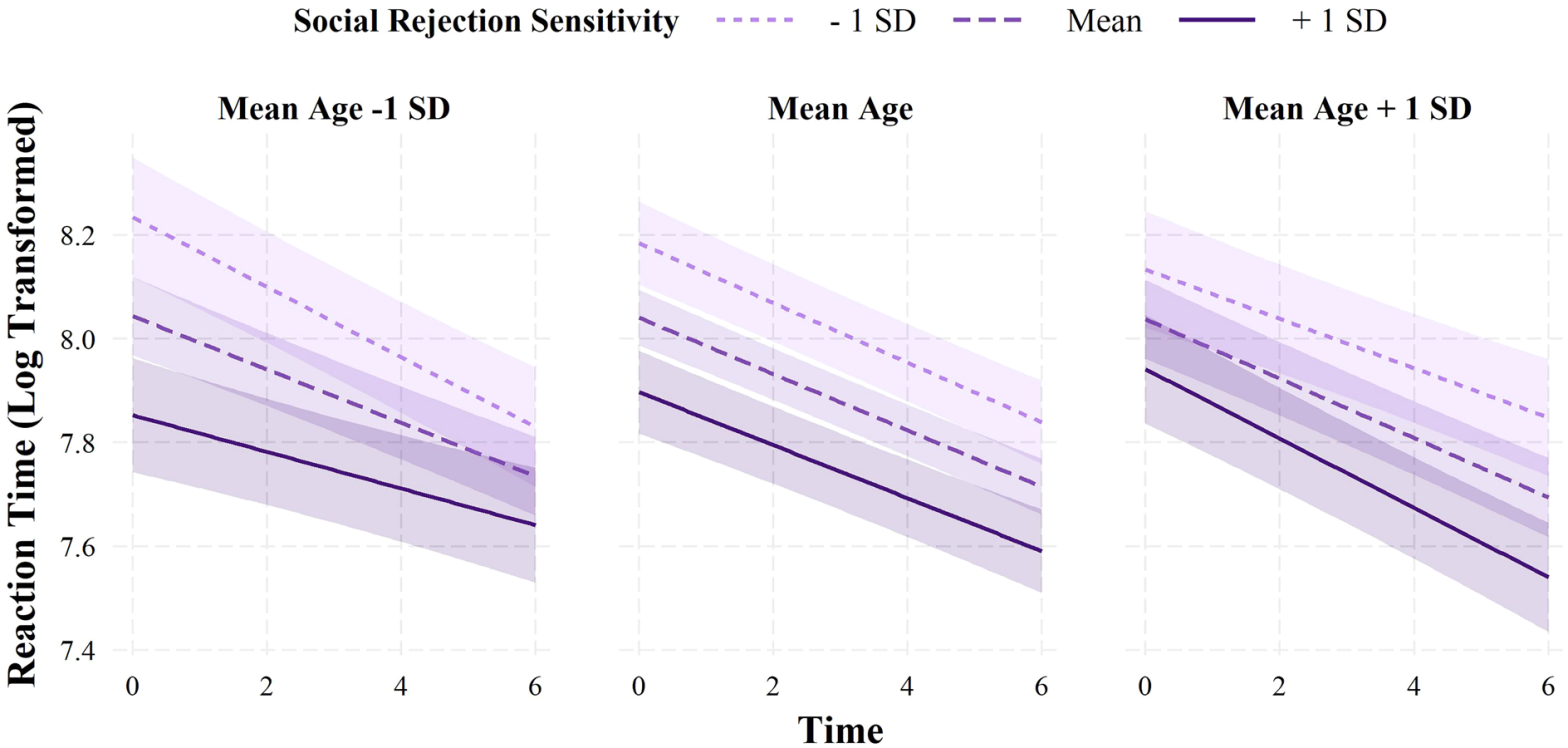
Learning rates on the ambiguous social scenarios task as a function of social rejection sensitivity and age. Time = time modelled as continuous variable indexing the seven themes on the ambiguous social scenarios task, with the first theme coded as 0; Social Rejection Sensitivity = social rejection sensitivity measured by the total score on the O^2^S^3^ [25]. social rejection sensitivityand age were mean-centred. Reaction time data were log transformed

Learning rates on the ASST were operationalized as change in reaction time and accuracy across the task (see Additional file 1: Table S3 for descriptives), analyzed using mixed effects models and generalized estimation equation models, respectively. When investigating H4, that learning rates would be associated with individual differences in SRS and negative interpretation bias, the reaction time analyses revealed conditional main effects of time, SRS, and negative interpretation bias (Additional file 1: Table S4). These conditional main effects were qualified by a significant three-way interaction between time, SRS, and age (*b* <  − 0.001, *SE* < 0.001, *t* =  − 3.60, *p* < 0.001; Fig. 3). Simple slopes analyses revealed that, amongst younger participants (1 *SD* below the mean), those with higher SRS (1 *SD* above the mean) showed less decrease in reaction time across the task (*b* =  − 0.04, *SE* = 0.01, *t* =  − 4.79, *p* < 0.001) compared to those with average (*b* =  − 0.05, *SE* = 0.01, *t* =  − 10.22, *p* < 0.001) and lower SRS (1 *SD* below the mean; *b* =  − 0.07, *SE* = 0.01, *t* =  − 8.82, *p* < 0.001). Amongst participants of average age, those with higher (1 *SD* above the mean; *b* =  − 0.05, *SE* = 0.01, *t* =  − 9.58, *p* < 0.001) and average SRS (*b* =  − 0.05, *SE* = 0.00, *t* =  − 15.31, *p* < 0.001), showed less decrease in reaction time compared to those with lower SRS (*b* =  − 0.06, *SE* = 0.01, *t* =  − 10.84, *p* < 0.001). Conversely, amongst older participants (1 *SD* above the mean), those with higher SRS (1 *SD* above the mean) showed a greater decrease in reaction time across the task (*b* =  − 0.07, *SE* = 0.01, *t* =  − 9.63, *p* < 0.001) compared to those with average (*b* =  − 0.06, *SE* = 0.01, *t* =  − 11.33, *p* < 0.001) and lower SRS (1 *SD* below the mean; *b* =  − 0.05, *SE* = 0.01, *t* =  − 6.44, *p* < 0.001). In an exploratory analysis, this interaction effect remained significant when controlling for emotional disorder symptoms. When examining the accuracy data, the analyses revealed significant conditional main effects of time (odds ratio = 1.12, *SE* = 0.03, *t* = 17.44, *p* < 0.001) and negative interpretation bias (odds ratio = 0.12, *SE* = 0.05, *t* = 23.56, *p* < 0.001; Additional file 1: Table S4). That is, accuracy on the task increased across time; however, was consistently lower amongst those with higher negative interpretation bias. These effects did not vary as a function of age.

**Fig. 4:**
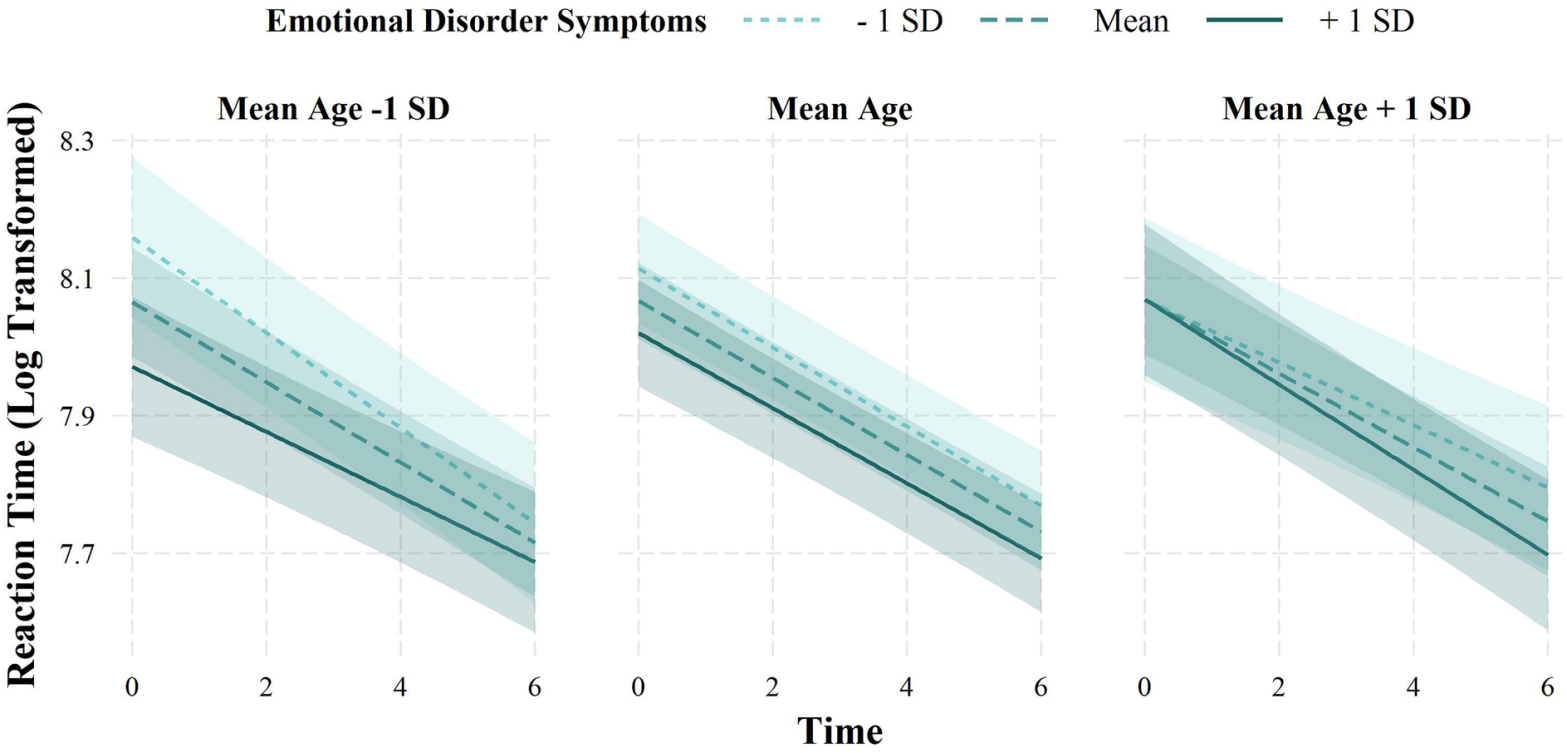
Learning rates on the ambiguous social scenarios task as a function of emotional disorder symptoms and age. Time = time modelled as continuous variable indexing the seven themes on the ambiguous social scenarios task, with the first theme coded as 0; emotional disorder symptoms = emotional disorder symptoms measured as composite score of standardized DASS-21 total score [30] and standardized SDQ internalizing score [31]. Emotional disorder symptoms and age were mean-centred. Reaction time data were log transformed

Separate models were specified to examine the influence of emotional disorder symptoms on learning rates on the ASST (H5). Again, the reaction time analyses revealed a significant conditional main effect of time, which was qualified by a significant 3-way interaction between time, emotional disorder symptoms, and age (*b* =  − 0.001, *SE* < 0.001, *t* =  − 2.69, *p* = 0.007; Additional file 1: Table S5; Fig. 4). Simple slopes analyses revealed that, amongst younger participants (1 *SD* below the mean), those with higher symptoms (1 *SD* above the mean) showed less decrease in reaction time across the task (*b* =  − 0.05, *SE* = 0.01, *t* =  − 7.13, *p* < 0.001) compared to those with average (*b* =  − 0.06, *SE* = 0.01, *t* =  − 11.61, *p* < 0.001) and lower symptoms (1 *SD* below the mean; *b* =  − 0.07, *SE* = 0.01, *t* =  − 9.50, *p* < 0.001). Amongst participants of average age, those with higher symptoms (1 *SD* above the mean; *b* =  − 0.05, *SE* = 0.01, *t* =  − 10.89, *p* < 0.001) showed less decrease in reaction time compared to those with average (*b* =  − 0.06, *SE* = 0.00, *t* =  − 15.92, *p* < 0.001) and lower symptoms (1 *SD* below the mean; *b* =  − 0.06, *SE* = 0.00, *t* =  − 11.55, *p* < 0.001). Conversely, amongst older participants (1 *SD* above the mean), those with higher symptoms (1 *SD* above the mean) showed a greater decrease in reaction time across the task (*b* =  − 0.06, *SE* = 0.01, *t* =  − 8.87, *p* < 0.001) compared to those with average (*b* =  − 0.05, *SE* = 0.00, *t* =  − 10.75, *p* < 0.001) and lower symptoms (1 *SD* below the mean; *b* =  − 0.05, *SE* = 0.01, *t* =  − 6.13, *p* < 0.001). When examining the accuracy data, the analyses revealed significant conditional main effects of time (odds ratio = 1.13, *SE* = 0.03, *t* = 21.03, *p* < 0.001) and emotional disorder symptoms (odds ratio = 0.72, *SE* = 0.04, *t* = 39.87, *p* < 0.001; Additional file 1: Table S5). That is, accuracy on the task increased across time; however, was consistently lower amongst those with higher symptoms. These effects did not vary as a function of age.

### The impact of social rejection sensitivity on change in negative interpretation bias following the ambiguous social scenarios task

**Fig. 5:**
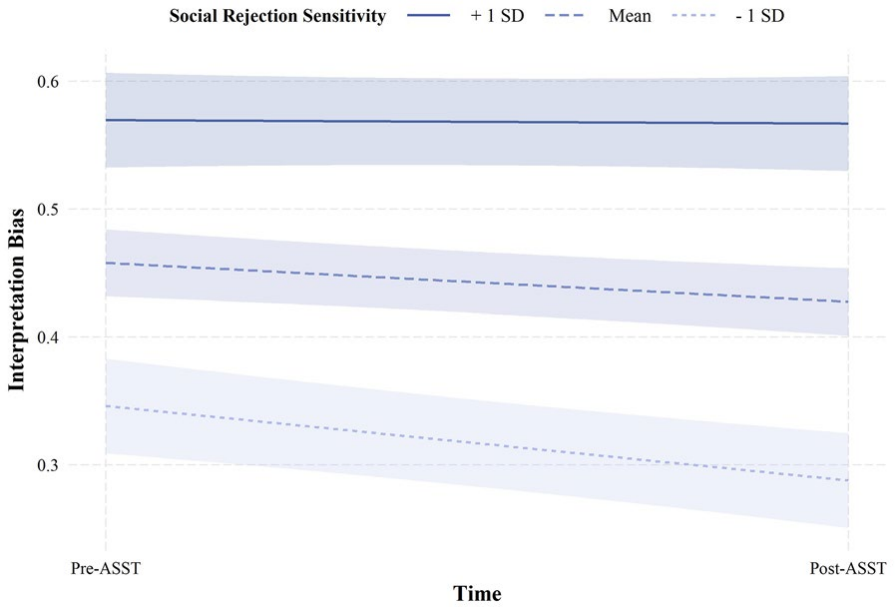
Change in negative interpretation bias from before to after the ambiguous social scenarios task as a function of social rejection sensitivity. Time = time modelled as dummy variable indexing the two administrations of the scrambled sentence task, before and after the ambiguous social scenarios task, with the pre administration coded as 0 and post administration coded as 1; Social rejection sensitivity = social rejection sensitivity measured by the total score on the O^2^S^3^ [25]; Interpretation bias = negative interpretation bias measured as proportion of grammatically correct sentences with a negative valence on the scrambled sentences task [23]

When examining change in negative interpretation bias from before to after the ASST, the analysis revealed conditional main effects of time and SRS (H6; Additional file 1: Table S8). These conditional main effects were qualified by a significant interaction between time and SRS (*b* = 0.003, *SE* = 0.001, *t* = 2.57, *p* = 0.010; Fig. 5). Simple slopes analyses revealed that those with low (1 *SD* below the mean) and average SRS demonstrated a decrease in negative interpretation bias following the task (*b* =  − 0.06, *SE* = 0.02, *t* =  − 3.81, *p* < 0.001; *b* =  − 0.03, *SE* = 0.01, *t* =  − 2.81, *p* = 0.005, respectively). Conversely, those with high SRS (1 *SD* above the mean) did not demonstrate a significant change in negative interpretation bias across time (*b* =  − 0.003, *SE* = 0.02, *t* =  − 0.18, *p* = 0.860). Thus, contrary to predictions, change in interpretation bias following the ASST *decreased* as a function of SRS. This effect did not vary as a function of age.

## Discussion

This study explored the role of SRS in emotional disorder symptoms during adolescence. In line with past research, results showed that SRS was strongly associated with negative interpretation bias and emotional disorder symptomatology in adolescents and young adults. A previously hypothesized antecedent of SRS, perceived parental rejection, was associated with heightened SRS while controlling for other types of perceived maladaptive parenting. Moreover, SRS was found to partially account for the variance between perceived parental rejection and emotional disorder symptomatology. SRS was further found to partially account for the variance between negative interpretation bias and emotional disorder symptomatology. The study’s second aim was to explore the role of SRS in individual differences in response to CBM-I. Rates of learning on a CBM-I task that required the positive resolution of socially ambiguous scenarios differed as a function of SRS and age; whereby amongst younger participants, higher SRS was associated with slower rates of learning on the task; conversely, amongst older participants, higher SRS was associated with faster rates of learning on the task. Moreover, individuals with greater SRS demonstrated less change in negative interpretation bias following the CBM-I task, regardless of age.

### The relationship between social rejection sensitivity, negative interpretation bias, perceived parental rejection and symptoms of emotional disorders in adolescents

The results of the current study suggest that SRS is strongly associated with symptoms of emotional disorders in adolescents. One pathway that may account for this association was through SRS’ association with negative interpretation bias. We found SRS to partially account for variance in the relationship between negative interpretation bias and emotional disorder symptomatology. Negative interpretation bias itself, like SRS, showed a strong association with emotional disorder symptomatology, consistent with the work implicating interpretation bias as a risk and maintaining factor for emotional disorders [15, 16], especially in socially ambiguous situations [5, 47, 48]. Indeed, Normansell & Wisco [49] found that negative interpretation bias fully mediated the relationship between SRS and depressive symptoms, suggesting that such a cognitive bias may play a mechanistic role in this relationship. While this may indeed be the case, the results of the current study point towards SRS and negative interpretation bias having partly separable roles in the experience of emotional disorder symptoms. We found significant indirect effects through both SRS and negative interpretation bias in the relationship between perceived parenting and emotional disorder symptoms. While the constructs are interrelated, and indeed, biased interpretation is referenced in the definition of SRS (i.e., the tendency to readily perceive rejection), there are components of SRS that do not map closely onto interpretation bias. For example, the emotional reaction that follows perceived or actual rejection may contribute to emotional disorder symptoms via a pathway other than interpretation bias, such as affective dysregulation [50].

Parenting style, and in particular, parental rejection, has been proposed as one antecedant of SRS. The current study’s findings support SRS theories arguing that experiences of rejection in important relationships contribute to heightened SRS, because the human need for belongingness and acceptance is too often met with rejection [5, 7]. Repeated rejection leads to these experiences becoming internalized and results in increased expectations of rejection as well as hypervigilance for signs of rejection in the future [5, 7]. The findings build upon current models by suggesting that SRS may in turn play a role in the relationship between perceived parental rejection and emotional difficulties. In line with these results, SRS (as well as emotion dysregulation, suppression and social withdrawal) was a significant mediator of the relationship between poor parenting and depression and trait anxiety symptoms [10]. Fewer studies have explored the relationship between parenting and negative interpretation bias, however, one study found that, amongst individuals with social phobia, a social developmental history marked by parental hostility was associated with negative interpretations of partner behaviour in a social interaction task [51]. Although longitudinal research is required to support this conclusion, our findings suggest that SRS and negative interpretation bias are potential mechanisms through which perceived parental rejection may lead to symptoms of emotional disorders in adolescents. A tendency to interpret ambiguous situations negatively, especially social situations, in turn, may lead to heightened dispositional SRS amongst adolescents, which may contribute to the heightened emotional disorder symptoms that characterizes adolescence. Future longitudinal research in which the serial mediation effect of parental rejection on emotional disorder symptoms, via negative interpretation bias and, in turn, SRS will help disentangle these effects.

Indeed, teaching effective and positive parenting techniques has been the focus of extensive research, funding and interventions [13]. The results of the current study support the utility of such interventions in potentially ameliorating mental health symptoms resulting from poor parenting. Nonetheless, parenting interventions tend to be costly and time-intensive. Thus, intervening at a later stage by, for example, targeting social-cognitive risk factors such as SRS and its behavioural correlate, negative interpretation bias, may prove fruitful.

### Learning to resolve ambiguous social scenarios positively: the role of individual differences

CBM-I is one such intervention that has shown to be effective in reducing interpretation bias, and in some cases, emotional disorder symptoms [19–21]. Cognitive behaviour therapy is the gold standard for treatment of emotional disorders, and it has been shown to successfully shift negative interpretation biases [52, 53]. Accessibility of such cognitive behaviour therapy, however, remains limited, with a recent review finding that as many as four out of five young people who could benefit from therapy are not accessing it, for reasons including stigma, costs and time demands, and geographic isolation [54]. Consequently, there is a need for alternative, time efficient, and easy to disseminate interventions.; CBM-I is one promising example of such an intervention [21].

In line with the CBM-I literature, the ASST successfully led to a decrease in interpretation bias, but only in individuals with low and average SRS. Those high on SRS demonstrated more entrenched negative interpretation bias. SRS theories posit that, in individuals with SRS, rejection experiences have become internalized, resulting in increased expectations of rejection as well as hypervigilance for signs of rejection in the future. Situations in which rejection is possible, such as ambiguous social situations, consequently activate this heightened anticipation of rejection [5, 13, 55]. In the current study, the ASST may have activated this heightened anticipation of rejection in those participants high on SRS, resulting in them readily perceiving rejection while responding to the post-scrambled sentences task. Due to the internalization of rejection experiences, then, negative interpretation bias in rejection sensitive individuals may be less easily modified.

Indeed, heightened SRS was associated with slower learning rates (i.e., less decrease in reaction time) on the ASST, but only amongst young adolescents (11 to 15 years). Conversely, amongst young adults, heightened SRS was associated with *increased* learning rates. These effects did not seem to be a consequence of mental health symptomatology in general, as the pattern of results held when emotional disorder symptoms were controlled for. Thus, a tendency to negatively interpret ambiguous social situations may be particularly entrenched in early adolescence. This fits with past research suggesting that SRS is highest in early adolescence [25, 56]. More generally, adolescence, and early adolescence in particular, is associated with heightened sensitivity to social cues [57]. Developmentally rapid and accurate processing of social information is critical to successfully navigate novel, often changing, social environments encountered in adolescence. Elevated SRS in this age group is therefore developmentally expected. Moreover, young adolescents have been shown to struggle to regulate affective responses to negative social information (e.g., images [58]) like the ones presented in the ASST). With increasing age, individuals may develop strategies that help them override prepotent negative interpretation tendencies, such as improved emotion regulation. It should be noted, however, that reaction time at the start of the task was fastest amongst those participants with higher SRS. In a similar vein, accuracy rates on the task were high from the beginning, and we did not observe an association between change in accuracy and SRS. Replication of these effects using a validated tool suitable for a wide age-range will therefore be important.

These findings may provide a possible explanation for why some previous studies have failed to observe transfer effects following CBM-I [21]. That is, individuals with high SRS, particularly early adolescents, may not benefit from CBM-I or may need multiple ‘doses’ of CBM-I to successfully modify their negative interpretation bias and facilitate transfer effects to mental health. Alternatively, perhaps the clinical utility of CBM-I could be increased by concurrently targeting distorted cognitions in rejection sensitive individuals. Future research in which individuals with high SRS receive multiple sessions of CBM-I will help to elucidate our findings. Such research would additionally allow us to determine whether interventions that target negative interpretation bias are also effective in reducing SRS.

## Strengths, limitations and directions for future research

The present study has several strengths, including a well-powered sample size, the inclusion of both behavioural and self-report measures, and the examination of constructs from adolescence to early adulthood. Moreover, the study method, hypotheses, and analysis plan were preregistered (https://osf.io/nwvs9). In addition, the sample of participants included in the present study was demographically diverse, with just over half identifying as female and less than half as White. This suggests that the findings may generalize across different genders and ethnicities. Nonetheless, the results of the present study should be interpreted in the context of the study’s limitations. First, the study was cross-sectional, thus preventing us from drawing any causal or directional inferences. For example, potential bi-directional relationships between SRS and negative interpretation bias could not be explored in the present data. Additionally, the study was conducted online and included self-report measures for key constructs. In particular, our measure of perceived parenting style was retrospective and responses may have been biased by participants’ current levels of SRS. That is, we cannot ascertain whether the present results reflect early parental rejection impacting on later SRS and emotional disorder symptomatology, or concurrent relationships between parental rejection with SRS and emotional disorder symptomatology. While the PASCQ has been used previously in studies with both adolescents and young adults [10], this questionnaire was designed for use by adolescents and may have been interpreted and responded to differently by adolescents and young adults. In particular as adolescents were reporting on their current parenting experiences, whereas young adults may have been reporting on past parenting experiences. While we attempted to control for age effects by including age as a covariate in analyses, possible differences in interpretation of questions by adolescents and young adults should be considered when interpreting the results of the present study. Moreover, we utilized a non-clinical sample and our age range was heavily distributed around the median. That is, we over-recruited adolescents, as we expected the adult age group to be more homogenous. The sample’s age distribution raises the possibility that study results may be more applicable to adolescents as opposed to young adults, and may also have prevented us from observing the expected associations between age and our variables of interest. Indeed, in contrast with previous research suggesting that SRS is greatest in adolescence [25, 59–61], the current study showed no significant association between SRS and age. The majority of adolescent participants were in the older end of the adolescent age range [2], whereas SRS appears highest in early adolescence [25, 56] The lack of age effect should therefore be replicated in a future study including more younger adolescents. Future research would also benefit from longitudinally exploring SRS across adolescence and adulthood.

Another potential limitation of the present study, as discussed previously, were the high accuracy rates on the ASST. That is, accuracy rates were very high from the start of the task therefore leaving little for improvement. Despite these high accuracy rates, participants nonetheless showed increased accuracy across the course of the ASST, indicating that some learning did occur on the task. As per our pre-registration, we included response time as an additional measure of learning on the task; with response time similarly showing, on average, a decrease (i.e., improvement/learning) across the course of the task. However, including response time on the ASST as a dependent variable has some limitations, as it is possible that taking more time on this task may in fact be an indication of greater engagement with the material or being slower to identify with positive interpretations; rather than a clear indication of negative interpretation bias. In addition, the ASST was developed specifically for the present study. While the design of the ASST was informed by commonly used CBM-I paradigms [21], the lack of prior validation of the task should be considered when interpreting the present results. The scenarios included in the ASST were also designed to be particularly relatable to adolescents, meaning that the present findings may be more applicable to adolescents as opposed to young adults.

Finally, the present study was administered completely online, meaning that participants completed the study without any supervision by researchers. It is therefore not possible to ensure that the questionnaires and tasks were completed independently by all participants.

## Conclusion

The present study explored the role of SRS in emotional disorder symptomatology in adolescents. The results of the study highlight promising targets for mental health interventions in adolescents. Specifically, SRS, as well as its antecedent parental rejection, and its behavioral correlate, negative interpretation bias, were strongly associated with the experience of emotional disorder symptoms. Importantly from a clinical perspective, high SRS was associated with reduced malleability of negative interpretation bias following a bias modification task, and, amongst early adolescents, slower learning rates on the task. Individual differences in SRS, then, especially amongst young adolescents, may partly account for the mixed findings on the effectiveness of interpretation-bias-modification paradigms. Future research is needed to investigate whether a higher dose of CBM-I may be effective in reducing the seemingly ingrained negative interpretation bias in individuals at risk for emotional disorders due to high SRS.

## Supplementary Information

**Additional file 1. **Participants. **2. **Cleaning ambiguous social scenarios task data. **3.** The relationship between social rejection sensitivity, negative interpretation bias, perceived parenting styles, and emotional disorder symptoms. **4.** The impact of social rejection sensitivity, negative interpretation bias, and emotional disorder symptomatology on learning rates on the ambiguous social scenarios task. **5. **The impact of social rejection sensitivity on change in negative interpretation bias following the ambiguous social scenarios task. **Table S1.** Social rejection sensitivity, negative interpretation bias, and emotional disorder symptomatology as a function of parenting styles. **Table S2.** Depression and anxiety symptoms as a function of parenting styles. **Table S3.** Descriptive statistics for reaction time and accuracy data on the ambiguous social scenarios task. **Table S4.** Learning rates on the ambiguous social scenarios task as a function of social rejection sensitivity, negative interpretation bias, and age. **Table S5.** Learning rates on the ambiguous social scenarios task as a function of emotional disorder symptoms and age. **Table S6.** Learning rates on the ambiguous social scenarios task as a function of depression symptoms and age. **Table S7.** Learning rates on the ambiguous social scenarios task as a function of anxiety symptoms and age. **Table S8.** Change in negative interpretation bias from before to after ambiguous social scenarios task as a function of social rejection sensitivity and age.

## Data Availability

The datasets used and/or analysed during the current study are available from the corresponding author on reasonable request.

## Abbreviations

AIC: Akaike Information Criterion
ASST: Ambiguous Social Scenarios Task
CBM-I: Cognitive Bias Modification for Interpretation
DASS-21: Depression Anxiety and Stress Scale-21
O^2^S^3^: Online and Offline Social Sensitivity Scale
PASCQ: Parents as Social Context Questionnaire
SDQ: Strengths and Difficulties Questionnaire
SES: Socioeconomic status
SRS: Social rejection sensitivity

## Footnotes

1 Our target sample size as per our pre-registration was 245. However, due to issues with online data collection (namely, the identification of a large number of duplicate participants) we recruited a larger sample of participants.

2 The Interpersonal Sensitivity Measure (IPSM [26]) was administered to validate the O^2^S^3^ in the present sample as the scale had not been published at the time of data collection for the present study. The IPSM supported the construct validity of the O^2^S^3^ in the present sample (*r* = 0.80).

3 A number of responses (12.04%) included less than five words. If these sentences were deemed to be grammatically correct, they were included in analyses.

4 As per our pre-registered method, we administered the affective backward digit span task [38]. This task requires participants to recall sequentially presented digits overlaid on neutral and affective images in reverse order. The affective backward digit span task was administered to ensure that any learning effects found on the ASST were not simply a function of differences in affective control. However, affective control showed no to small (−0.13 to −0.01) correlations with the variables of interest, and was consequently not included as a covariate in analyses.
