# Supplementary material for: Social rejection sensitivity and its role in adolescent emotional disorder symptomatology
Source: Child Adolesc Psychiatry Ment Health. 2023 Jan 16;17:8. doi: 10.1186/s13034-022-00555-x (PMC9843960; doi:10.1186/s13034-022-00555-x)
Supplement: Supplementary file 1 — Additional file 1. Participants. 2. Cleaning ambiguous social scenarios task data. 3. The relationship between social rejection sensitivity, negative interpretation bias, perceived parenting styles, and emotional disorder symptoms. 4. The impact of social rejection sensitivity, negative interpretation bias, and emotional disorder symptomatology on learning rates on the ambiguous social scenarios task. 5. The impact of social rejection sensitivity on change in negative interpretation bias following the ambiguous social scenarios task. Table S1. Social rejection sensitivity, negative interpretation bias, and emotional disorder symptomatology as a function of parenting styles. Table S2. Depression and anxiety symptoms as a function of parenting styles. Table S3. Descriptive statistics for reaction time and accuracy data on the ambiguous social scenarios task. Table S4. Learning rates on the ambiguous social scenarios task as a function of social rejection sensitivity, negative interpretation bias, and age. Table S5. Learning rates on the ambiguous social scenarios task as a function of emotional disorder symptoms and age. Table S6. Learning rates on the ambiguous social scenarios task as a function of depression symptoms and age. Table S7. Learning rates on the ambiguous social scenarios task as a function of anxiety symptoms and age. Table S8. Change in negative interpretation bias from before to after ambiguous social scenarios task as a function of social rejection sensitivity and age. [file 13034_2022_555_MOESM1_ESM.docx]

**Social Rejection Sensitivity and its Role in Adolescent Emotional Disorder Symptomatology**

**Additional file 1**

**1: Participants**

Fifteen participants were excluded due to answering more than one attention item incorrectly or missing data on attention items; five participants were excluded due to reporting an age greater than 30; four participants were excluded due to reporting a history of TBI; four participants were excluded due to reporting a history of neurodevelopmental/neurological disorder; one participant was excluded due to a lack of English fluency; 76 duplicate participants were excluded; and one participant was excluded due to answering more than one attention item incorrectly, and reporting a history of TBI and a history of neurodevelopmental/neurological disorder.

**2: Cleaning Ambiguous Social Scenarios Task Data**

First, data were filtered to include only trials in which participants were required to choose between a positive and negative resolution (i.e., neutral filler trials were removed), resulting in four trials per seven themes. Next, reaction times on trials in which participants responded correctly (i.e., selected the positive resolution) were collated and theme average reaction times were computed. Similarly, average accuracy (i.e., proportion of positive resolutions selected) was calculated for each theme.

In order to identify outlier data, sample average reaction time and standard deviation were computed. Participants’ theme reaction time and corresponding accuracy averages were excluded if their mean theme reaction time was below 500ms, which is below the time it would minimally take to read the scenario and resolution options. Additionally, extreme values that were 3 standard deviations above or below the sample mean reaction time were also excluded. Inspection of the cleaned accuracy data, however, revealed left-skewed distributions (skew ranged from −1.06 to −1.79; kurtosis ranged from 0.16 to 2.30), such that the majority of participants were performing at ceiling on this task (i.e., answering 100% of trials correctly on each theme). The cleaned reaction time data similarly showed poor skew (ranging from 1.01 to 1.57) and kurtosis (ranging from 1.44 to 4.03) values. Consequently, the accuracy data was transformed into a binary distribution (100% correct or less than 100% correct) and the binary accuracy data was subsequently submitted to a generalized estimation equation model. The cleaned reaction time data was log transformed and submitted to a linear mixed effects model, with a random effect for participant ID.

**3: The Relationship Between Social Rejection Sensitivity, Negative Interpretation Bias, Perceived Parenting Styles, and Emotional Disorder Symptoms**

**Table S1**

*Social Rejection Sensitivity, Negative Interpretation Bias, and Emotional Disorder Symptomatology as a Function of Parenting Styles*

|  | **SRS** | | | | **Interpretation Bias** | | | | | **Emotional Disorder Symptomatology** | | | | |
| --- | --- | --- | --- | --- | --- | --- | --- | --- | --- | --- | --- | --- | --- | --- |
| *Coefficient* | *b* | *SE* | *t-value* | *p-value* | *b* | | *SE* | *t-value* | *p-value* | *b* | | *SE* | *t-value* | *p-value* |
| Intercept | 16.51 | 3.63 | 4.55 | **<0.001** | 0.21 | | 0.08 | 2.54 | **0.012** | −2.79 | | 0.62 | −4.51 | **<0.001** |
| Parental Rejection | **5.90** | **1.59** | **3.70** | **<0.001** | **0.08** | | **0.04** | **2.21** | **0.028** | **0.99** | | **0.27** | **3.71** | **<0.001** |
| Parental Behavioural Control | −1.63 | 1.48 | −1.10 | 0.274 | 0.02 | | 0.03 | 0.61 | 0.543 | 0.09 | | 0.24 | 0.39 | 0.695 |
| Parental Psychological Control | 2.50 | 1.59 | 1.57 | 0.117 | 0.04 | | 0.04 | 1.12 | 0.262 | 0.37 | | 0.26 | 1.42 | 0.158 |
| Age | −0.02 | 0.15 | −0.16 | 0.873 | −0.00 | | 0.00 | −0.54 | 0.589 | −0.01 | | 0.03 | −0.24 | 0.812 |
| Observations | 266 | | | | | 287 | | | | | 259 | | | |
| R^2^ / R^2^ adjusted | 0.213 / 0.201 | | | | 0.151 / 0.139 | | | | | 0.286 / 0.275 | | | | |

*Note.* SRS = social rejection sensitivity measured by the total score on the O^2^S^3^ [25]; interpretation bias = negative interpretation bias measured as proportion of grammatically correct sentences with negative valence on the pre-ASST scrambled sentences task [23]; emotional disorder symptomatology = emotional disorder symptoms measured as composite score of standardized DASS-21 total score [30] and standardized SDQ internalizing score [31]; parental rejection = perceived parental rejection measured as eponymous subscale score of the PASCQ [36]; parental behavioural control = perceived parental behavioural control measured as eponymous subscale score of the PASCQ [36]; parental psychological control = perceived parental psychology control measured as eponymous subscale score of the PASCQ [36].

Perceived parental rejection was found to be associated with heightened depression and anxiety symptoms, while controlling for perceived parental psychological control and behavioural control (SM Table 2). Conversely, perceived parental psychological and behavioural control were not associated with depression and anxiety symptoms, while controlling for perceived parental rejection.

**Table S2**

*Depression and Anxiety Symptoms as a Function of Parenting Styles*

|  | **Depression Symptoms** | | | | **Anxiety Symptoms** | | | |
| --- | --- | --- | --- | --- | --- | --- | --- | --- |
| *Coefficient* | *b* | *SE* | *t-value* | *p-value* | *b* | *SE* | *t-value* | *p-value* |
| Intercept | −2.93 | 3.76 | −0.78 | 0.436 | 1.64 | 3.51 | 0.47 | 0.640 |
| Parental Rejection | **6.85** | **1.64** | **4.18** | **<0.001** | **6.08** | **1.55** | **3.91** | **<0.001** |
| Parental Behavioural Control | −0.89 | 1.52 | −0.59 | 0.557 | 1.00 | 1.42 | 0.70 | 0.482 |
| Parental Psychological Control | 2.91 | 1.61 | 1.81 | 0.071 | −0.62 | 1.52 | −0.41 | 0.685 |
| Age | 0.10 | 0.15 | 0.63 | 0.527 | −0.10 | 0.14 | −0.70 | 0.484 |
| Observations | 283 |  |  |  | 279 |  |  |  |
| R^2^ / R^2^ adjusted | 0.274 / 0.264 | | | | 0.187 / 0.175 | | | |

*Note.* Depression and anxiety symptoms = depression and anxiety symptoms measured as eponymous subscale scores of the DASS-21 [30]; parental rejection = perceived parental rejection measured as eponymous subscale score of the PASCQ [36]; parental behavioural control = perceived parental behavioural control measured as eponymous subscale score of the PASCQ [36]; parental psychological control = perceived parental psychological control measured as eponymous subscale score of the PASCQ [36].

The relationship between perceived parental rejection and depression symptoms was partially accounted for by SRS (standardized indirect effect: *β* = 0.13, *SE* = 0.04, *z* = 3.92, *p* < 0.001) and negative interpretation bias (standardized indirect effect: *β* = 0.10, *SE* = 0.04, *z* = 4.02, *p* < 0.001); AIC = 2272.40). The relationship between parental rejection and anxiety symptoms was partially accounted for by SRS (standardized indirect effect: *β* = 0.18, *SE* = 0.05, *z* = 5.28, *p* < 0.001) but not by negative interpretation bias (standardized indirect effect: *β* = 0.03, *SE* = 0.03, *z* = 1.21, *p* = 0.225); AIC = 2315.96).

SRS partially accounted for the relationship between negative interpretation bias and depression symptoms (standardized indirect effect: *β* = 0.17, *SE* = 0.13, *z* = 5.40, *p* < 0.001; AIC = 1522.66) and the relationship between negative interpretation bias and anxiety symptoms (standardized indirect effect: *β* = 0.21, *SE* = 0.14, *z* = 6.09, *p* < 0.001; AIC = 1553.09).

**4: The Impact of Social Rejection Sensitivity, Negative Interpretation Bias, And Emotional Disorder Symptomatology on Learning Rates on the Ambiguous Social Scenarios Task**

**Table S3**

*Descriptive Statistics for Reaction Time and Accuracy Data on the Ambiguous Social Scenarios Task*

| **Time** | **Reaction Time**  *M (SD); range* | **Accuracy**  *M (SD); range* |
| --- | --- | --- |
| **1** | 3849.35 (2084.99); 581.00:11593.27 | 0.83 (0.21); 0.25:1.00 |
| **2** | 3203.13 (1697.85); 534.17:9637.00 | 0.85 (0.22); 0.25:1.00 |
| **3** | 2960.30 (1594.74); 538.88:10183.00 | 0.87 (0.22); 0.25:1.00 |
| **4** | 2741.30 (1358.97); 519.69:10219.84 | 0.87 (0.22); 0.25:1.00 |
| **5** | 2821.78 (1563.84); 503.00:10928.00 | 0.87 (0.22); 0.25:1.00 |
| **6** | 2685.80 (1409.75); 518.13:9737.00 | 0.88 (0.21); 0.25:1.00 |
| **7** | 2662.84 (1449.7); 563.67:10677.50 | 0.89 (0.20); 0.25:1.00 |

*Note*. SM Table 3 shows sample descriptive statistics for reaction time and accuracy across the seven themes of the ASST. Reaction Time = reaction time in milliseconds, measured as average response time (for positive resolutions) for each theme. Accuracy = accuracy measured as proportion of positive resolutions selected within each theme. These sample descriptive statistics exclude outlier responses, which were defined as reaction times below 500ms and extreme values that were 3 standard deviations above or below the sample mean reaction time (see SM2 for further details).

**Table S4**

*Learning Rates on the Ambiguous Social Scenarios Task as a Function of Social Rejection Sensitivity, Negative Interpretation Bias, and Age*

|  | **Reaction Time** | | | | **Accuracy** | | | |
| --- | --- | --- | --- | --- | --- | --- | --- | --- |
| *Coefficient* | *b* | *SE* | *t-value* | *p-value* | *Odds Ratios* | *SE* | *t-value* | *p-value* |
| Intercept | 8.04 | 0.03 | 294.64 | **<0.001** | 1.66 | 0.15 | 30.61 | **<0.001** |
| Time | **−0.05** | **0.00** | **−15.32** | **<0.001** | **1.12** | **0.03** | **17.44** | **<0.001** |
| SRS | **−0.01** | **0.00** | **−4.71** | **<0.001** | 0.98 | 0.01 | 3.71 | 0.054 |
| Age | −0.00 | 0.01 | −0.10 | 0.917 | 0.98 | 0.02 | 0.94 | 0.331 |
| Bias | **0.51** | **0.12** | **4.12** | **<0.001** | **0.12** | **0.05** | **23.56** | **<0.001** |
| Time x SRS | 0.00 | 0.00 | 0.81 | 0.420 | 1.00 | 0.00 | 1.72 | 0.190 |
| Time x Age | −0.00 | 0.00 | −0.91 | 0.361 | 1.00 | 0.01 | 0.05 | 0.823 |
| SRS x Age | 0.00 | 0.00 | 1.71 | 0.087 | 1.00 | 0.00 | 2.27 | 0.132 |
| Time x Bias | −0.02 | 0.02 | −1.46 | 0.145 | 1.12 | 0.14 | 0.76 | 0.384 |
| Age x Bias | −0.03 | 0.03 | −1.26 | 0.210 | 1.17 | 0.11 | 2.56 | 0.109 |
| Time x SRS x Age | **−0.00** | **0.00** | −**3.60** | **<0.001** | 1.00 | 0.00 | 1.29 | 0.256 |
| Time x Bias x Age | 0.00 | 0.00 | 0.64 | 0.521 | 0.98 | 0.03 | 0.40 | 0.527 |
| **Random Effects** |  |  |  |  |  |  |  |  |
| σ^2^ | 0.10 | | | |  | | | |
| τ_00_ | 0.17 _ID_ | | | |  | | | |
| ICC | 0.63 | | | |  | | | |
| N | 286 _ID_ | | | | 286 _ID_ | | | |
| Observations | 1918 | | | | 1918 | | | |
| Marginal R2 / Conditional R2 | 0.103 / 0.672 | | | |  | | | |

*Note*. Time = time modelled as continuous variable indexing the seven themes on the ASST, with the first theme coded as 0; SRS = social rejection sensitivity measured by the total score on the O^2^S^3^ [25]; Bias = negative interpretation bias measured as the proportion of grammatically correct sentences with negative valence on the pre-ASST scrambled sentences task [23]. SRS, bias, and age were mean-centred. Reaction time data is on a log scale and accuracy data is on a binary distribution, where 1 = 100% correct and 0 = < 100% correct.

**Table S5**

*Learning Rates on the Ambiguous Social Scenarios Task as a Function of Emotional Disorder Symptoms and Age*

|  | **Reaction Time** | | | | **Accuracy** | | | |
| --- | --- | --- | --- | --- | --- | --- | --- | --- |
| *Coefficient* | *b* | *SE* | *t-value* | *p-value* | *Odds Ratios* | *SE* | *t-value* | *p-value* |
| Intercept | 8.06 | 0.03 | 283.62 | <0.001 | 1.56 | 0.14 | 23.75 | <0.001 |
| Time | **−0.06** | **0.00** | **−15.80** | **<0.001** | **1.13** | **0.03** | **21.03** | **<0.001** |
| Emotional Disorder Symptoms | −0.02 | 0.02 | −1.56 | 0.119 | **0.72** | **0.04** | **39.87** | **<0.001** |
| Age | 0.00 | 0.01 | 0.17 | 0.862 | 1.00 | 0.02 | 0.01 | 0.940 |
| Time x Emotional Disorder Symptoms | 0.00 | 0.00 | 0.24 | 0.808 | 0.99 | 0.02 | 0.66 | 0.416 |
| Time x Age | 0.00 | 0.00 | 0.46 | 0.642 | 1.00 | 0.01 | 0.01 | 0.932 |
| Emotional Disorder Symptoms x Age | 0.01 | 0.00 | 1.64 | 0.100 | 0.99 | 0.01 | 1.01 | 0.314 |
| Time x Emotional Disorder Symptoms x Age | **−0.00** | **0.00** | **−2.69** | **0.007** | 1.01 | 0.00 | 2.99 | 0.084 |
| **Random Effects** |  | | | |  | | | |
| σ^2^ | 0.09 | | | |  | | | |
| τ_00_ | 0.18 _ID_ | | | |  | | | |
| ICC | 0.66 | | | |  | | | |
| N | 280 _ID_ | | | | 280 _ID_ | | | |
| Observations | 1875 | | | | 1875 | | | |
| Marginal R^2^ / Conditional R^2^ | 0.053 / 0.680 | | | |  | | | |

*Note*. Time = time modelled as continuous variable indexing the seven themes on the ASST, with the first theme coded as 0; emotional disorder symptoms = emotional disorder symptoms measured as composite score of standardized DASS-21 total score [30] and standardized SDQ internalizing score [31]. Emotional disorder symptoms and age were mean-centred. Reaction time data is on a log scale and accuracy data is on a binary distribution, where 1 = 100% correct and 0 = < 100% correct.

When investigating the impact of depression symptoms on learning rates on the ASST, the reaction time analyses revealed a significant conditional main effect of time (SM Table 6). When examining the accuracy data, the analyses revealed significant conditional main effects of time and depression symptoms (SM Table 6). That is, accuracy on the task increased across time; however, was consistently lower amongst those with higher depressive symptoms. These effects did not vary as a function of age.

**Table S6**

*Learning Rates on the Ambiguous Social Scenarios Task as a Function of Depression Symptoms and Age*

|  | **Reaction Time** | | | | **Accuracy** | | | |
| --- | --- | --- | --- | --- | --- | --- | --- | --- |
| *Coefficient* | *b* | *SE* | *t-value* | *p-value* | *Odds Ratios* | *SE* | *t-value* | *p-value* |
| Intercept | 8.01 | 0.03 | 284.97 | **<0.001** | 1.64 | 0.15 | 28.13 | **<0.001** |
| Time | **−0.05** | **0.00** | **−15.66** | **<0.001** | **1.13** | **0.03** | **20.14** | **<0.001** |
| Depression Symptoms | −0.01 | 0.00 | −2.31 | **0.021** | **0.95** | **0.01** | **50.09** | **<0.001** |
| Age | −0.00 | 0.01 | −0.22 | 0.828 | 1.00 | 0.02 | 0.02 | 0.902 |
| Time x Depression Symptoms | 0.00 | 0.00 | 0.24 | 0.807 | 1.00 | 0.00 | 0.15 | 0.694 |
| Time x Age | 0.00 | 0.00 | 0.11 | 0.909 | 1.00 | 0.01 | 0.08 | 0.778 |
| Depression Symptoms x Age | −0.00 | 0.00 | −0.23 | 0.815 | 1.00 | 0.00 | 0.19 | 0.666 |
| Time x Depression Symptoms x Age | −0.00 | 0.00 | −0.39 | 0.699 | 1.00 | 0.00 | 4.00 | **0.045** |
| **Random Effects** | | | | | | | | |
| σ^2^ | 0.10 | | | |  | | | |
| τ_00_ | 0.20 _ID_ | | | |  | | | |
| ICC | 0.68 | | | |  | | | |
| N | 314 _ID_ | | | | 280 _ID_ | | | |
| Observations | 2099 | | | | 1875 | | | |
| Marginal R^2^ / Conditional R^2^ | 0.050 / 0.692 | | | |  | | | |

*Note*. Time = time modelled as continuous variable indexing the seven themes on the ASST, with the first theme coded as 0; depression symptoms = depression symptoms measured as eponymous subscale score of the DASS-21 [30]. Depression symptoms and age were mean-centred. Reaction time data is on a log scale and accuracy data is on a binary distribution, where 1 = 100% correct and 0 = < 100% correct.

When investigating the impact of anxiety symptoms on learning rates on the ASST, the reaction time analyses revealed significant conditional main effects of time and anxiety symptoms (SM Table 7). These main effects were qualified by a significant 3-way interaction between time, anxiety symptoms, and age. Simple slopes analyses revealed that, amongst younger participants (1 *SD* below the mean), those with higher anxiety symptoms (1 *SD* above the mean) showed less decrease in reaction time across the task (*b* = −0.04, *SE* = 0.01, *t* = −6.25, *p* < 0.001) compared to those with average (*b* = −0.06, *SE* = 0.00, *t* = −11.22, *p* < 0.001) and lower symptoms (1 *SD* below the mean; *b* = −0.07, *SE* = 0.01, *t* = −10.10, *p* < 0.001). Amongst participants of average age, those with higher (1 *SD* above the mean; *b* = −0.05, *SE* = 0.00, *t* = −9.46, *p* < 0.001) and average symptoms (*b* = −0.05, *SE* = 0.00, *t* = −15.48, *p* < 0.001) showed less decrease in reaction time compared to those with lower symptoms (*1* SD below the mean; *b* = −0.06, *SE* = 0.00, *t* = 12.43, *p* < 0.001). Conversely, amongst older participants (1 *SD* above the mean), change in reaction time did not differ as a function of anxiety symptoms (1 *SD* above the mean: *b* = −0.05, *SE* = 0.01, *t* = −8.20, *p* < 0.001; average: *b* = −0.05, *SE* = 0.00, *t* = −10.33, *p* < 0.001; 1 *SD* below the mean; *b* = −0.05, *SE* = 0.01, *t* = −6.77, *p* < 0.001). When examining the accuracy data, the analyses revealed significant conditional main effects of time and anxiety symptoms (SM Table 7). That is, accuracy on the task increased across time; however, was consistently lower amongst those with higher anxiety symptoms. These effects did not vary as a function of age.

**Table S7**

*Learning Rates on the Ambiguous Social Scenarios Task as a Function of Anxiety Symptoms and Age*

|  | **Reaction Time** | | | | **Accuracy** | | | | |
| --- | --- | --- | --- | --- | --- | --- | --- | --- | --- |
| *Coefficient* | *b* | *SE* | *t-value* | *p-value* | *Odds Ratios* | | *SE* | *t-value* | *p-value* |
| Intercept | 8.00 | 0.03 | 291.52 | **<0.001** | 1.57 | | 0.14 | 24.73 | **<0.001** |
| Time | **−0.05** | **0.00** | **−15.36** | **<0.001** | **1.13** | | **0.03** | **21.56** | **<0.001** |
| Anxiety Symptoms | **−0.01** | **0.00** | **−5.83** | **<0.001** | **0.95** | | **0.01** | **29.11** | **<0.001** |
| Age | −0.01 | 0.01 | −1.21 | 0.228 | 0.99 | | 0.02 | 0.17 | 0.682 |
| Time x Anxiety Symptoms | 0.00 | 0.00 | 2.01 | **0.044** | 1.00 | | 0.02 | 1.10 | 0.293 |
| Time x Age | 0.00 | 0.00 | 0.55 | 0.579 | 1.00 | | 0.01 | 0.02 | 0.879 |
| Anxiety Symptoms x Age | −0.00 | 0.01 | −0.63 | 0.527 | 1.00 | | 0.00 | 3.24 | 0.072 |
| Time x Anxiety Symptoms x Age | **−0.00** | **0.00** | **−3.05** | **0.002** | 1.00 | | 0.00 | 1.41 | 0.235 |
| **Random Effects** | | | | | | | | | |
| σ^2^ | 0.10 | | | | |  | | | |
| τ_00_ | 0.19 _ID_ | | | | |  | | | |
| ICC | 0.66 | | | | |  | | | |
| N | 312 _ID_ | | | | | 280 _ID_ | | | |
| Observations | 2083 | | | | | 1875 | | | |
| Marginal R^2^ / Conditional R^2^ | 0.106 / 0.694 | | | | |  | | | |

*Note*. Time = time modelled as continuous variable indexing the seven themes on the ASST, with the first theme coded as 0; anxiety symptoms = anxiety symptoms measured as eponymous subscale score of the DASS-21 [30]. Anxiety symptoms and age were mean-centred. Reaction time data is on a log scale and accuracy data is on a binary distribution, where 1 = 100% correct and 0 = < 100% correct.

**5: The Impact of Social Rejection Sensitivity on Change in Negative Interpretation Bias following the Ambiguous Social Scenarios Task**

**Table S8**

*Change in Negative Interpretation Bias from Before to After Ambiguous Social Scenarios Task as a Function of Social Rejection Sensitivity and Age*

|  | **Negative Interpretation Bias** | | | |
| --- | --- | --- | --- | --- |
| *Coefficient* | *b* | *SE* | *t-value* | *p-value* |
| Intercept | 0.46 | 0.01 | 34.39 | <0.001 |
| Time | **−0.03** | **0.01** | **−2.79** | **0.005** |
| SRS | **0.01** | **0.00** | **8.35** | **<0.001** |
| Age | −0.00 | 0.00 | −0.87 | 0.385 |
| Time x SRS | **0.00** | **0.00** | **2.57** | **0.010** |
| Time x Age | −0.01 | 0.00 | −1.96 | 0.051 |
| SRS x Age | −0.00 | 0.00 | −1.72 | 0.086 |
| Time x SRS x Age | 0.00 | 0.00 | 0.15 | 0.880 |
| **Random Effects** | | | | |
| σ^2^ | 0.02 | | | |
| τ_00_ | 0.03 _ID_ | | | |
| ICC | 0.67 | | | |
| N | 293 | | | |
| Observations | 578 | | | |
| Marginal R^2^ / Conditional R^2^ | 0.252 / 0.757 | | | |

*Note*. Time = time modelled as dummy variable indexing the two administrations of the scrambled sentence task, before and after the ambiguous social scenarios task, with the first administration coded as 0; SRS = social rejection sensitivity measured by the total score on the O^2^S^3^ [25]; Negative interpretation bias = negative interpretation bias measured as the proportion of grammatically correct sentences with negative valence on the pre-ASST scrambled sentences task [23]. SRS and age were mean-centred.
